# 6-Bromoisatin Found in Muricid Mollusc Extracts Inhibits Colon Cancer Cell Proliferation and Induces Apoptosis, Preventing Early Stage Tumor Formation in a Colorectal Cancer Rodent Model

**DOI:** 10.3390/md12010017

**Published:** 2013-12-24

**Authors:** Babak Esmaeelian, Catherine A. Abbott, Richard K. Le Leu, Kirsten Benkendorff

**Affiliations:** 1 School of Biological Sciences, Flinders University, GPO Box 2100, Adelaide 5001, Australia; E-Mails: esma0007@flinders.edu.au (B.E.); cathy.abbott@flinders.edu.au (C.A.); 2 Flinders Centre for Innovation in Cancer, Flinders University, GPO Box 2100, Adelaide 5001, Australia; E-Mail: Richard.leleu@flinders.edu.au; 3 Preventative Health National Research Flagship, CSIRO, PO Box 10041, Adelaide BC 5000, Australia; 4 Marine Ecology Research Centre, School of Environment, Science and Engineering, Southern Cross University, GPO Box 157, Lismore, NSW 2480, Australia

**Keywords:** apoptosis, azoxymethane, bioactive natural product, colorectal cancer, isatin, *in vivo* model, marine mollusc

## Abstract

Muricid molluscs are a natural source of brominated isatin with anticancer activity. The aim of this study was to examine the safety and efficacy of synthetic 6-bromoisatin for reducing the risk of early stage colorectal tumor formation. The purity of 6-bromoisatin was confirmed by ^1^H NMR spectroscopy, then tested for *in vitro* and *in vivo* anticancer activity. A mouse model for colorectal cancer was utilized whereby colonic apoptosis and cell proliferation was measured 6 h after azoxymethane treatment by hematoxylin and immunohistochemical staining. Liver enzymes and other biochemistry parameters were measured in plasma and haematological assessment of the blood was conducted to assess potential toxic side-effects. 6-Bromoisatin inhibited proliferation of HT29 cells at IC_50_ 223 μM (0.05 mg/mL) and induced apoptosis without increasing caspase 3/7 activity. *In vivo* 6-bromoisatin (0.05 mg/g) was found to significantly enhance the apoptotic index (*p* ≤ 0.001) and reduced cell proliferation (*p* ≤ 0.01) in the distal colon. There were no significant effects on mouse body weight, liver enzymes, biochemical factors or blood cells. However, 6-bromoisatin caused a decrease in the plasma level of potassium, suggesting a diuretic effect. In conclusion this study supports 6-bromoisatin in Muricidae extracts as a promising lead for prevention of colorectal cancer.

## 1. Introduction

Isatin (1*H*-indole-2,3-dione) is a synthetically versatile molecule, and its derivatives possess diverse biological and pharmacological properties, including antibacterial, antifungal, antiviral, anticonvulsant, and anticancer activities [1,2,3,4,5]. Isatin itself has demonstrated cytotoxic and apoptotic activity. For example, Cane *et al.* [6] showed that isatin at a concentration of 100 µM reduced cell proliferation of human promyelocytic leukemia (HL60) cancer cells by 80% and induced morphological changes consistent with proapoptotic cells (including DNA fragmentation and chromatin condensation). In another study by Igosheva *et al.* [7], apoptosis was observed in human neuroblastoma SH-SY5Y cells exposed to 50 µM of isatin. A range of mono-substituted isatins have been studied by Vine *et al.* [8] for their *in vitro* cytotoxicity on a lymphoma (U937) cell line. Structure activity relationship studies have shown that substitution with halogens (5-bromo-, 5-iodo-, and 5-fluoroisatin) yielded 5–10 times more activity for killing cancer cells, than the unsubstituted isatin [8]. Sunitinib (Sutent^®^) is a fluorinated isatin derivative that has been approved by FDA as a new anticancer drug to treat advanced renal carcinoma [9] and gastrointestinal stromal tumors [10].

**Figure 1:**
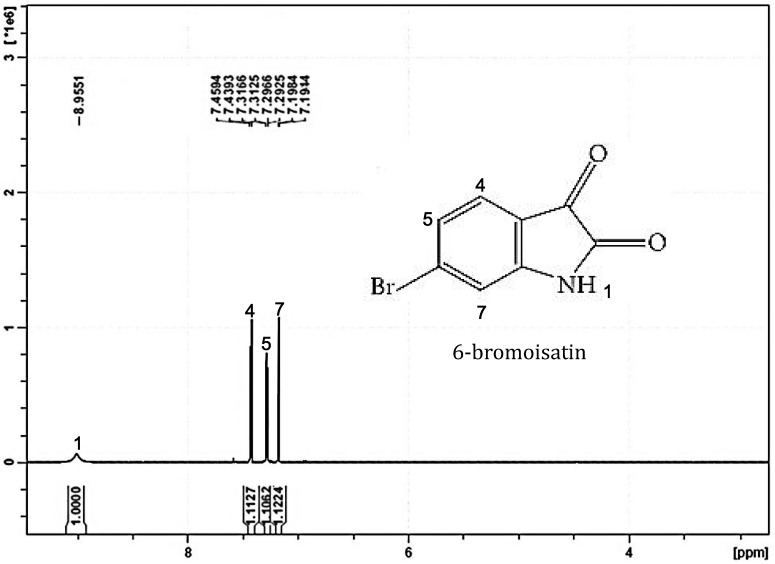
^1^H NMR spectrum of synthetic 6-bromoisatin on the Bruker Avance III 400 MHz spectrometer in deuterated acetonitrile. Chemical shifts (δ) are as parts per million (ppm) and referenced to residual solvent peaks. The peaks corresponding to the solvent occur below 3 ppm are not shown.

Various substituted isatins have been found in nature including in plants [11], fungi [12] and marine molluscs [5,13]. Recently 6-bromoisatin (Figure 1) from the Australian marine mollusc *Dicathais orbita* has become of particular interest as a major compound of the bioactive extract from this species [5]. In a study by Edwards *et al.* [14], semi-purified 6-bromoisatin from *D*. *orbita* extracts revealed specific anticancer activity with >10 fold selective cytotoxicity towards female reproductive cancer cells compared to freshly isolated human granulosa cells. Furthermore, semi-purified 6-bromoisatin was shown to significantly reduce proliferation and induce apoptosis in human colon cancer cell lines HT29 and Caco2 cells [15]. In a short-term rodent model for the prevention of colon cancer Westley *et al.* [16] demonstrated that the crude extract of *D. orbita* increased the apoptotic index of distal colon cells significantly. Due to several contaminants in the naturally purified extract [15,16], further work using the pure synthesized compound is required to confirm the activity of 6-bromoisatin against colon cancer cells.

Colorectal cancer (CRC) is the third most common cancer worldwide [17] with the highest incidence rates in Australia, New Zealand, North America and Europe [18]. In the United States, CRC is the second highest cause of cancer-related mortality in both males and females [19]. Just 39% of CRCs are diagnosed at early stage and in most cases the cancer spreads to adjacent and distant organs before detection [20]. Therefore, prevention of CRC is an important priority [21]. Chemoprevention involves the use of functional foods, specific natural products or synthetic chemical agents to suppress or prevent a wide range of cancers, including colon cancer [22]. The acute apoptotic response to genotoxic carcinogens (AARGC) is a good model for chemopreventative research which has been used in several studies [16,23,24,25,26,27]. In this model, the carcinogen azoxymethane (AOM) is injected into mice causing DNA damage in epithelial cells in the crypts of the distal colon inducing an acute apoptotic response 6–8 h later. The AARGC model has been mainly used to identify the effect of natural products on inducing apoptosis of the damaged colon cells, with the aim of detecting early stage CRC prevention [25].

In a previous *in vivo* study using a two-week preventative treatment with the crude extract from *D. orbita*, we detected an increase in apoptosis in the colon of mice in response to AOM injection. The aim of this study is to test the *in vitro* and *in vivo* effects of pure synthetic 6-bromoisatin, to confirm whether this compound is the key factor in *D. orbita* extracts responsible for the inhibition of colon cancer cells and the induction of apoptosis in damaged colon cells in the AARGC rodent model of colon cancer prevention. We also obtained additional data to assess any potential side effects of synthetic 6-bromoisatin on blood parameters and liver toxicity in the mice.

## 2. Results and Discussion

### 2.1. Chemical Analysis

^1^H NMR results showed four major peaks corresponding to the four hydrogen protons in the 6-bromoisatin molecule: ^1^H NMR (400 MHz, CD3CN) δ 8.96 (^1^H, s), 7.44 (^1^H, d, *J* = 8.08 Hz), 7.30 (^1^H dd, *J* = 1.64, 8 Hz), 7.19 (^1^H, d, *J* = 1.6 Hz) and confirming the identity of synthetic 6-bromoisatin and its high purity (Figure 1). The ^1^H NMR spectra for synthetic 6-bromoisatin matches our previous NMR data for semi-purified 6-bromoisatin in anticancer extracts from the marine mollusc *D. orbita* [15].

### 2.2. *In Vitro* Apoptosis, Necrosis and Cell Viability

**Figure 2:**
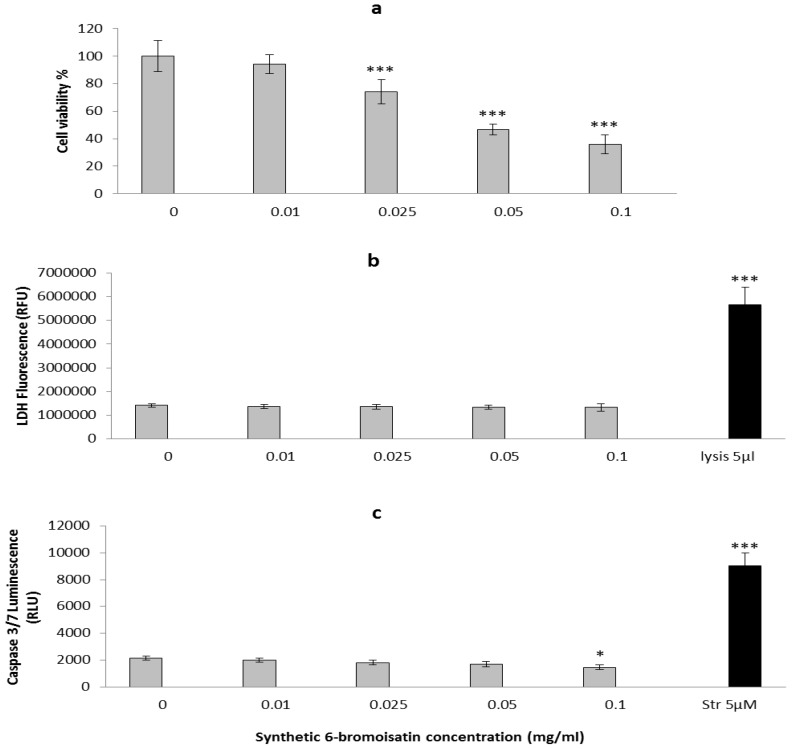
Effects of synthetic 6-bromoisatin on HT29 cells: Cell viability (**a**), LDH release (**b**) and caspase-3/7 activity (**c**). LDH release was measured by fluorescence at 535EX/590EM and caspase-3/7 activity was measured at full light on a luminescence plate reader. The positive controls are lysis buffer (5 µL/well) for the LDH assay and staurosporine (Str) (5 µM) for apoptosis. A final concentration of 1% DMSO was used in all control and treated cells. The results are mean for three independent repeat assays (*n* = 3) each performed in triplicate. Significant differences between each group and the DMSO control are shown as *p* ≤ 0.05 (*) and *p* ≤ 0.001 (***).

The effects of 6-bromoisatin on proliferation, apoptosis and necrosis of HT29 cells was examined. A dose dependent effect of 6-bromoisatin on the viability of cells was observed using the 3-(4,5-dimethylthiazol-2-yl)-2 5-diphenyltetrazolium bromide (MTT) assay for inhibition of metabolic activity (Figure 2). The three highest concentrations of 6-bromoisatin (1 mg/mL, 0.05 mg/mL and 0.025 mg/mL) significantly reduced the cell viability by 64%, 53% and 26% respectively (*p* < 0.001), relative to the DMSO control, but no significant reduction was observed at the lowest dose of 0.01 mg/mL (*p* > 0.05). The IC_50_ for synthetic 6-bromoisatin was calculated at 223 μM (0.05 mg/mL) for HT29 cells (Figure 2a). However, our previous *in vitro* study using semi-purified 6-bromoistain, on both HT29 and Caco2 cells, revealed a lower IC_50_ of 100 μM [15], suggesting possible synergistic activity with other factors in the extract. The *in vitro* cytotoxic effects of synthetic 6-bromoisatin in this study could also be due to lower bioavailability of the pure compound to the cells when compared to the natural extract, which contains trace lipids that may help dissolve this lipophylic compound and/or facilitate interactions with cell membrane lipids. Previous studies have reported lower bioavailability of some synthetic compounds, in comparison with the naturally purified compounds [28,29]. For example, the bioavailability ratio of natural Vitamin E *versus* synthetic Vitamin E was shown to be close to 2:1 [29], which is similar to our study.

**Figure 3:**
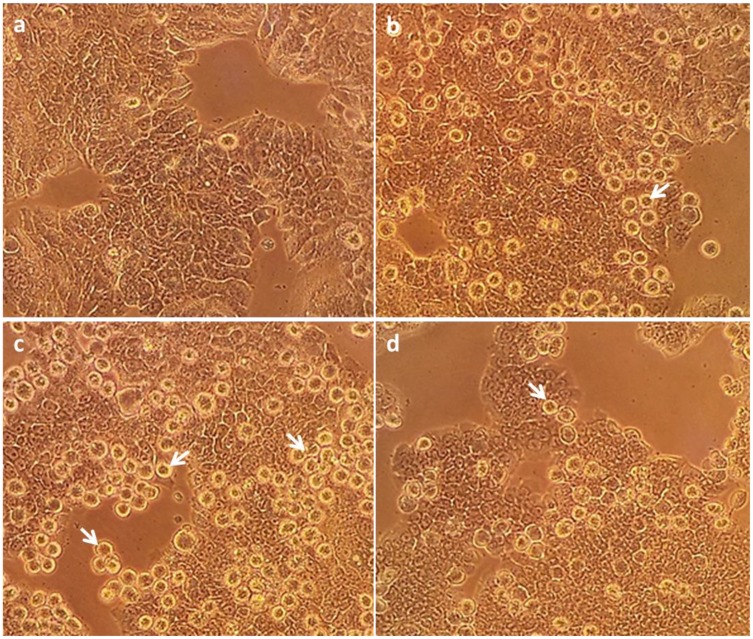
HT29 cells at 400× magnification under the Olympus inverted microscope. One percent dimethylsulphoxide (DMSO) control (**a**); cells treated with 0.025 mg/mL synthetic 6-bromoisatin (**b**); cells treated with 0.05 mg/mL synthetic 6-bromoisatin (**c**) and cells treated with 0.1 mg/mL synthetic 6-bromoisatin (**d**) for 12 h (final concentration of 1% DMSO). Examples of apoptotic cells with chromatin condensation and surrounded by a halo are indicated by the arrows.

No increase in the level of lactate dehydrogenase (LDH) (Figure 2b) a measure of necrosis or late stage apoptosis was observed in the cells treated with any concentration of 6-bromoisatin, in comparison with DMSO control. Unexpectedly, synthetic 6-bromoisatin did not increase caspase 3/7 activity in HT29 cells *in vitro* at concentrations <0.1 mg/mL. This is in conflict with our previous study on semi-purified 6-bromoisatin, which significantly upregulated caspase 3/7 activity in HT29 cells [15]. The positive controls, lysis buffer and staurosporine, resulted in a significant increase in LDH activity (Figure 2b, *p* < 0.001) and caspase 3/7 activity (Figure 2c, *p* <0.001) respectively, demonstrating that the assays were working. The cells treated with the highest concentration of 6-bromoisatin (446 μM = 0.1 mg/mL) showed a minor but significant reduction of caspase3/7 activity compared to the DMSO control (Figure 2c, *p* = 0.011). Nevertheless, the light microscopic images from the HT29 cells treated with 223 μM and 112 μM 6-bromoisatin showed morphological alterations, such as chromatin condensation, characteristic of the apoptotic process (Figure 3b,c). Apoptotic cells were also observed in cultures treated with the highest dose of 6-bromoisatin 446 μM, although in lower numbers than the two other doses (Figure 3d). This indicates that synthetic 6-bromoisatin may induce apoptosis in the HT29 cells through a caspase-independent pathway. In the past few years, the existence of caspase-independent programmed cell death pathways have been reported in the literature, which are associated with executioners other than the caspases, such as cathepsins, calpains, serine proteases and also apoptosis inducing factor (AIF) protein [30].

In terms of the mode of action, the isatin molecule has been proposed to interact via extracellular signal regulated protein kinases (ERKs) to inhibit cancer cell proliferation and promote apoptosis [2]. In a study by Cane *et al.* [6], isatin at a concentration of 100 µM inhibited the phosphorylation of ERK-2 (but not ERK-1) by 35% compared to the control. ERK is attributed to a survival signaling pathway in several cell types; however, it mediates apoptosis in some cell types and organs (e.g., neuronal and renal epithelial cells) under certain conditions [31]. Although the mechanisms for mediating apoptosis by ERK is not fully understood, three mechanisms have been proposed: (1) ERK1/2 may act through the intrinsic apoptotic pathway by up-regulating Bax and p53 followed by mitochondrial cytochrome c release and activation of caspase-3 [31,32,33]; (2) Through the extrinsic pathway by increasing an upstream signal for death receptors, such as TNF-α followed by activation of caspase-8 and caspase-3 [31,34] or; (3) Through inhibition of Akt (Protein kinase B) mediated survival signaling [35]. As synthetic 6-bromoisatin in this study induced apoptosis in HT29 cells without activating caspase-3 and considering the fact that both intrinsic and extrinsic pathways are associated with upregulation of caspase-3, the third pathway resulting in a decrease in Akt activity is hypothesized as a caspase-independent apoptosis pathway for synthetic 6-bromoisatin. However, further mode of action studies that specifically target Akt gene expression in colon cancer cells are required with 6-bromoisatin to confirm this.

### 2.3. *In Vivo* Mouse Model

#### 2.3.1. Mice; General Observations

Given synthetic 6-bromoisatin reduced the cell viability of a colon cancer cell line *in vitro*, we tested its effects on the AARGC response in a mouse model of CRC. The mice did not show any signs of illness in the treatment groups or the control group during the study. The body weights of all mice increased steadily over the trial duration, without any significant differences in mean total weight gain between the treatment groups and the control (Table 1, *p* = 0.999). No significant change in the liver weight or the percentage liver to body weight (*p* = 0.098) was revealed between treatment groups and the control (Table 1).

**Table 1:** Comparison of mean (±S.E.) progressive body weight (g) in controls and mice treated with different concentrations of 6-bromoisatin on different days of the experiment. All treatments and the control were injected with 10 mg/kg AOM 6 h prior to kill. Liver weight (g) and percentage liver weight/body weight were calculated on the day of kill. *n* = 10 mice in treatment groups and *n* = 8 mice in the control.

Weight (g)							
Concentration	Body (Day1)	Body (Day5)	Body (Day10)	Body (Day14)	Total weight Gain	Liver	Liver/Body (%)
**Control**	22.0 ± 1.6	22.6 ± 1.6	22.8 ± 1.9	23.5 ± 1.9	1.4 ± 0.7	1.1 ± 0.3	4.8 ± 1.2
**0.025 mg/g**	22.2 ± 1.3	22.7 ± 1.3	23.0 ± 1.3	23.6 ± 1.4	1.4 ± 0.8	1.0 ± 0.1	4.4 ± 0.6
**0.05 mg/g**	22.6 ± 1.2	23.4 ± 1.2	23.4 ± 1.5	24.1 ± 1.6	1.4 ± 1.0	1.2 ± 0.1	5.2 ± 0.6
**0.1 mg/g**	22.3 ± 1.3	22.6 ± 1.3	23.3 ± 1.3	23.8 ± 1.6	1.4 ± 0.8	1.3 ± 0.1	5.3 ± 0.4

#### 2.3.2. Apoptotic Index, Crypt Height and Cell Proliferation

**Figure 4:**
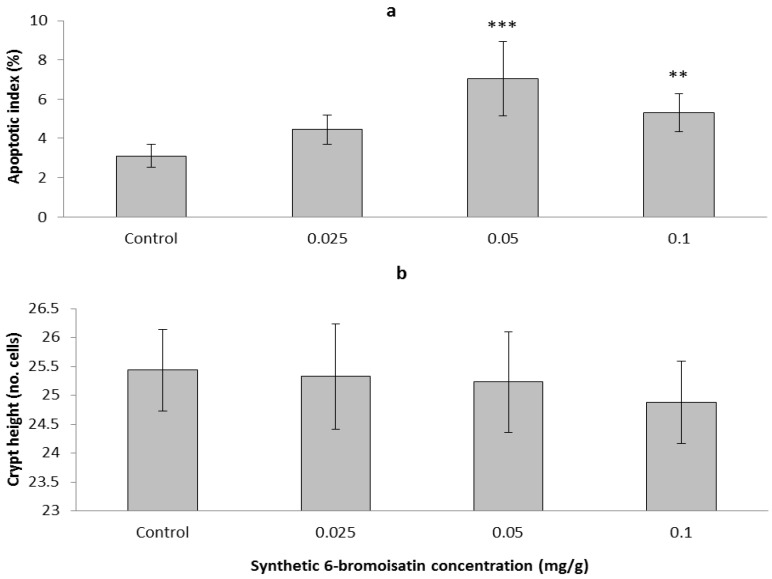
Apoptotic response and crypt height in the distal colon of mice after 14 day oral gavage with different concentrations of synthetic 6-bromoisatin, showing: apoptotic index (**a**) and crypt height (**b**). All treatments and the oil only control were injected with 10 mg/kg AOM 6 h prior to kill. Data are means ± S.E. for 10 full crypts/animal (*n* = 10 mice in treatment groups and *n* = 8 mice in control group). Significant differences between each group and the control are shown as *p* ≤ 0.01 (**) and *p* ≤ 0.001 (***).

**Figure 5:**
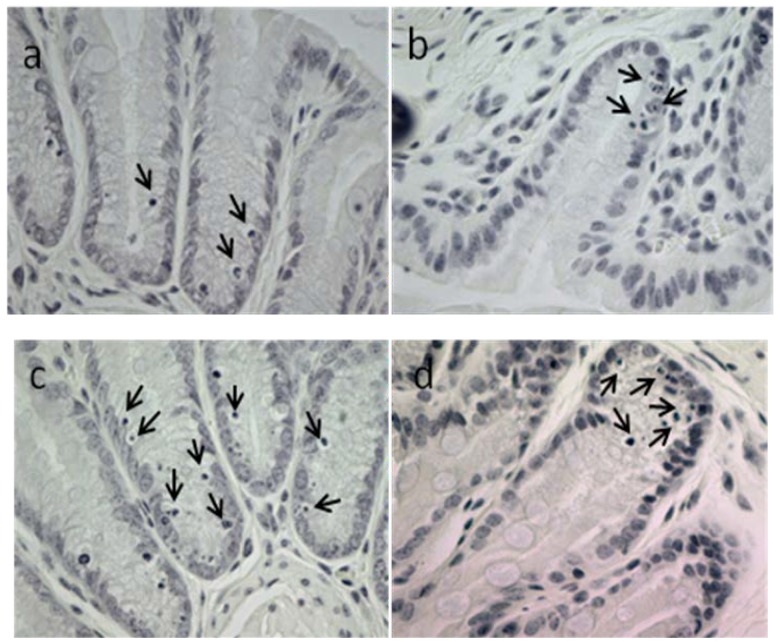
Apoptosis in the basal crypt cells of the distal colon of mice 6 h post AOM injection (10 mg/kg). Mice were oral gavaged daily for two weeks prior with oil. (**a**) control; (**b**) 6-bromoisatin 0.025 mg/g; (**c**) 6-bromoisatin 0.05 mg/g; (**d**) 6-bromoisatin 0.1 mg/g. Apoptotic cells with chromatin condensation characteristics are shown by arrows.

Synthetic 6-bromoisatin was found to significantly increase apoptosis in response to AOM injection (ANOVA *F* = 14.660, *p* < 0.001, *df* = 3), but had no significant effect on colon crypt height (ANOVA *F* = 1.013, *p* = 0.403, *df* = 3); (Figure 4). The mice treated for two weeks daily with 0.05 mg/g 6-bromoisatin showed the greatest increase in apoptotic index in the distal colon (Figure 4a), with a 2.3 fold increase over the oil control (*p* ≤ 0.001). The highest dose of 6-bromoisatin (0.1 mg/g) also significantly induced apoptosis (*p* = 0.007) in the distal colon of the mice compared with the control group. However, this effect was significantly lower, by 40%, than the dose of 0.05 mg/g (*p* = 0.031). In contrast, although the distal colon of the mice administered with the lowest concentration of 6-bromoisatin showed a slightly increased apoptosis index, there was not a significant difference when compared to the AOM injected control (Figure 4a, *p* = 0.158). Apoptosis in the distal colon of the mice occurred mostly in basal crypt cells (Figure 5).

**Figure 6:**
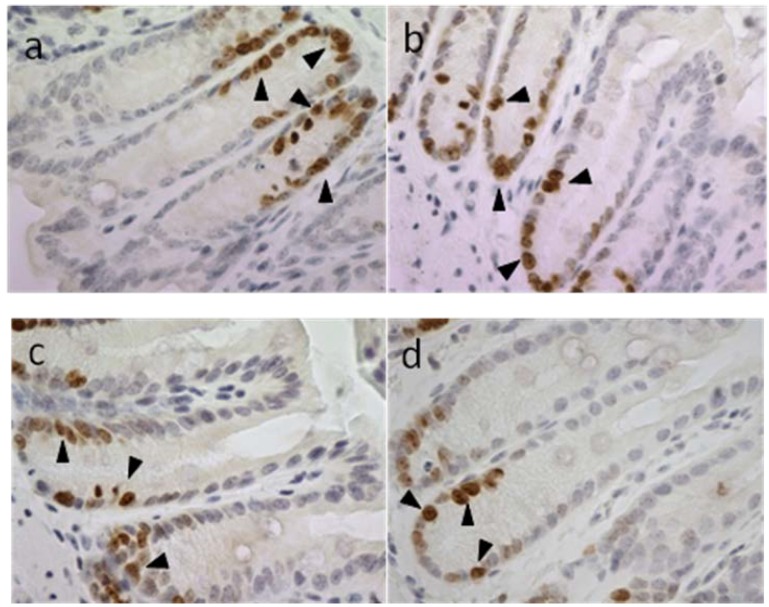
Proliferative activity of distal colonic epithelial cells in mice 6 h after AOM injection (10 mg/kg), shown using an antibody specific for the ki-67 antigen. Mice were oral gavaged daily for two weeks prior with oil; (**a**) control; (**b**) 6-bromoisatin 0.025 mg/g; (**c**) 6-bromoisatin 0.05 mg/g; (**d**) 6-bromoisatin 0.1 mg/g. Proliferating cells are shown by arrowheads.

**Figure 7:**
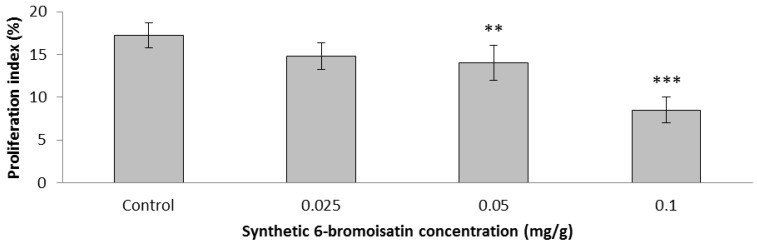
Proliferation index in the distal colon of oil control mice and mice treated with different concentrations of 6-bromoisatin by daily oral gavage for 2 weeks, followed by 10 mg/kg AOM injection 6 h prior to kill. Significant differences between each group and the control are shown as *p* ≤ 0.01 (**) and *p* ≤ 0.001 (***).

Ki-67 immunohistochemistry showed evidence for cell proliferation, also in the basal cells of colon crypts of mice, in response to AOM injection (Figure 6). After two weeks daily oral gavage, synthetic 6-bromoisatin was found to significantly reduce this cell proliferation in the distal colon of mice (ANOVA *F* = 41.273, *p* < 0.001, *df* = 3); (Figure 7). After AOM injection, the mice treated with the highest concentration of 6-bromoisatin (0.1 mg/g) had the greatest reduction in cell proliferation in the distal colon, by more than 50% compared to control mice gavaged with oil alone (*p* ≤ 0.001). Similarly, the dose of 0.05mg/g significantly reduced the proliferation in the distal colon compared to the oil alone control (*p* = 0.006), and was not significantly different to the higher dose of 0.1 mg/g (*p* = 0.652). In contrast, the lowest dose of 6-bromoisatin (0.025 mg/g) did not show a significant anti-proliferative effect in the distal colon as compared to the oil alone control (Figure 6 and Figure 7, *p* = 0.052).

A connection between AARGC stimulation and the inhibition of oncogenesis in the distal colon has been previously shown in mice [36,37]. In our study, synthetic 6-bromoisatin at the concentration of 0.05 mg/g had the greatest effect in facilitating apoptosis in the distal colon of mice. Interestingly, the apoptotic index at the highest dose of synthetic 6-bromoisatin (0.1 mg/g) was lower than the middle dose of 0.05 mg/g, which is consistent with a previous study using the crude extract from *D. orbita* [16]. This could be related to the fact that cell proliferation in the colon showed the highest reduction at the highest dose of 0.1 mg/g 6-bromoisatin, indicating that fewer DNA damaged cells may have been present that required removal by the initiation of programmed cell death. In a study by Saini *et al.* [38] the cytotoxic effect of streptozotocin, at low doses, was shown to be associated with the activation of the apoptotic pathway on beta cells, whereas this predominantly changed to necrosis at high doses. Therefore the lower apoptosis index with the highest dose of 6-bromoisatin may indicate that some necrosis or cell cycle arrest occurred in the crypt cells at this high dose. However, the *in vitro* LDH assays found no evidence of an increase in cell membrane permeability that would suggest necrosis at this concentration.

#### 2.3.3. Blood Biochemistry and Hematology

**Figure 8:**
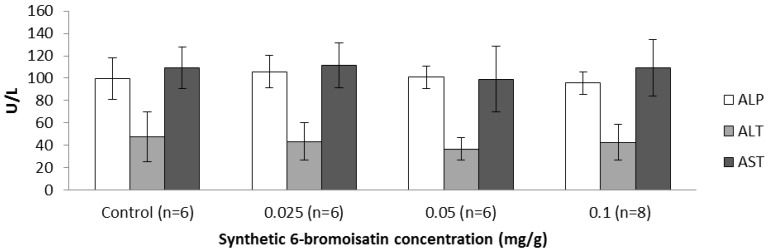
Liver enzymes *aspartate aminotransferase* (AST), *alanine aminotransferase* (ALT) and *alkaline phosphatase* (ALP) levels in serum (U/L) of oil control mice and mice treated with different concentrations of 6-bromoisatin by daily oral gavage for two weeks.

To be useful as a future drug or nutraceutical for the prevention of colon cancer, 6-bromoisatin and/or *D. orbita* extracts must also be safe for oral use. The plasma level of the liver enzymes aspartate aminotransferase (AST), alanine aminotransferase (ALT) and alkaline phosphatase (ALP) are indicators of hepatotoxicity [39]. No significant difference was revealed in the plasma level of these enzymes in the oil control, as compared to treatment groups gavaged for two weeks daily with 6-bromoisatin (Figure 8, *p* > 0.6), providing evidence that synthetic 6-bromoisatin is not hepatotoxic at these concentrations. In a study by Westley *et al.* [40], mice treated with the crude *D. orbita* extracts containing 6-bromoisatin showed some idiosyncratic toxicity in the liver, whereas pure 6-bromoisatin did not exhibit this hepatotoxicity.

In the plasma there were no significant differences in sodium, urea, creatinine, calcium, protein, albumin and globulin levels in the mice treated with 6-bromoisatin in comparison to the oil control (Table 2; *p* > 0.05). However, a significant dose dependent reduction in potassium plasma levels (Hypokalemia) was observed in the mice administered with both 0.05 mg/g (*p* = 0.005) and 0.1 mg/g 6-bromoisatin (*p* = 0.001), as compared to the oil control (Table 2). Consequently, the sodium/potassium ratio (Na/K) increased significantly in these groups compared to the oil control (Table 2, *p* < 0.005).

The most common reason for a potassium deficiency is diuretic therapy (loop diuretics, thiazides) that causes urinary potassium excretion [41,42], and gastrointestinal potassium wasting from diarrhea [41,43]. In this study, no diarrhea or change in stool consistency was observed in either the oil control or 6-bromoisatin treatment groups. Therefore, the potassium deficiency in the treatment groups might be due to a diuretic effect of 6-bromoisatin leading to an increase in urinary potassium excretion. Diuretic effects of some novel isatin derivatives, especially the derivatives of bromoisatin, were previously shown by Nataraj *et al.* [44]. Consequently, future *in vivo* studies using 6-bromoisatin should carefully monitor this possible diuretic effect.

**Table 2:** Plasma biochemistry and blood hematology from mice in the oil only control group and mice treated with different concentrations of 6-bromoisatin, 6 h after injection of 10 mg/kg AOM. Significant differences between each group and the oil control are shown as *p* ≤ 0.05 (*), *p* ≤ 0.01 (**) and *p* ≤ 0.001 (***). Significant differences between 6-bromoistain doses are shown as *p* ≤ 0.05 (^#^) relative to 0.1 mg/mL. Na/K = Sodium/Potassium ratio, Creat = Creatinine, Hct = Hematocrit, MCV = Mean Corpuscular Volume, MCH = Mean Corpuscular Hemoglobin, MCHC = Mean Corpuscular Hemoglobin Concentration.

	Oil Control (*n* = 6)	6-Bromoisatin (0.025 mg/g, *n* = 6)	6-Bromoisatin (0.05 mg/g, *n* = 6)	6-Bromoisatin (0.1 mg/g, *n* = 8)
**Biochemistry**				
**Sodium (mmol/L)**	146.5 ± 1.2	147.7 ± 0.5	149.7 ± 1.4	149.7 ± 1.2
**Potassium (mmol/L)**	5.4 ± 0.3	5.1 ± 0.2 ^#^	4.5 ± 0.4 *	4.3 ± 0.4 **
**NA/K**	27.0 ± 2.2	29.1 ± 1.2 ^#^	33.5 ± 2.7 **	34.1 ± 3.6 ***
**Urea (mmol/L)**	10.6 ± 1.2	9.7 ± 1.6	9.9 ± 1.4	9.6 ± 1.2
**Creat. (umol/L)**	14.2 ± 1.2	13.3 ± 0.8	14.7 ± 0.5	14.6 ± 1.2
**Calcium (mmol/L)**	2.2 ± 0.03	2.2 ± 0.1	2.2 ± 0.04	2.2 ± 0.1
**Protein (g/L)**	45.8 ± 3.0	46.7 ± 2.8	46.2 ± 1.9	46.1 ± 2.2
**Albumin (g/L)**	28.2 ± 1.7	28.7 ± 1.5	27.8 ± 1.5	28.2 ± 1.5
**Globulin (g/L)**	17.7 ± 1.5	18.0 ± 1.4	18.3 ± 0.8	17.9 ± 1.2
**Hematology**				
**Red cell count (×10^12^/L)**	9.0 ± 0.4	9.3 ± 0.4	9.1 ± 0.4	9.4 ± 0.5
**Hemoglobin (g/L)**	135.2 ± 2.7	139.7 ± 5.1	135.0 ± 3.5	138.4 ± 5.7
**Hct (L/L)**	0.4 ± 0.01	0.4 ± 0.01	0.4 ± 0.01	0.4 ± 0.02
**MCV (FL)**	46.0 ± 1.2	46.5 ± 0.8	46.7 ± 1.2	46.4 ± 0.7
**MCH (Pg)**	15.2 ± 0.4	15.0 ± 0.0	15.0 ± 0.0	15.0 ± 0.0
**MCHC (g/L)**	323.7 ± 4.5	322.7 ± 1.5	319.0 ± 2.8	319.8 ± 3.0
**White cell count (×10^9^/L)**	4.8 ± 0.9	5.2 ± 1.8	5.8 ± 1.5 ^#^	3.2 ± 1.1
**Neutrophils (×10^9^/L)**	1.9 ± 0.3	1.6 ± 0.8	2.2 ± 1.3	0.8 ± 0.7
**Lymphocytes (×10^9^/L)**	2.8 ± 0.9	3.3 ± 1.1	3.4 ± 0.8	2.3 ± 0.9
**Monocytes (×10^9^/L)**	0.2 ± 0.1	0.2 ± 0.3	0.2 ± 0.1	0.1 ± 0.1

The hematological factors including white blood count, red blood count, hemoglobin, hematocrit (Hct), mean corpuscular volume (MCV), mean corpuscular hemoglobin (MCH), mean corpuscular hemoglobin concentration (MCHC), band form neutrophils, lymphocytes and monocytes were not significantly altered in treatment groups, in comparison to the oil only control (Table 2, *p* > 0.05). However, there was a significant increase in the white blood cell count of mice treated with 0.05 mg/g 6-bromoistain, compared to mice treated with the higher dose of 0.1 mg/g (Table 2, *p* = 0.037). This may indicate some mild anti-inflammatory effects at the higher dose of 6-bromoistain. Isatin has been previously found to inhibit NO production, COX-2, TNF and PGE2 in mouse macrophages [45]. Furthermore, indirubin derivatives exhibit inflammatory activity in RAW 264.7 cells [46] and in rat brain microglia [47].

Overall, this study demonstrates that 6-bromoisatin, a dominant compound found in muricid mollusc extract, has anticancer effects and low toxicity *in vivo*. Pure synthetic 6-bromoisatin effectively reduced the proliferation of colon cells, both *in vitro* against the HT29 colorectal cancer cell line and *in vivo* in mice administered AOM, which causes DNA damage in colon cells. 6-Bromoisatin also enhanced the apoptotic response in DNA damaged colon cells *in vivo*, with 0.05 mg/g found to be the most effective dose, with the only sign of toxicity after two weeks administration being a possible diuretic effect. Although synthetic 6-bromoisatin did not increase caspase 3/7 activity in HT29 cells, light microscopy confirmed the presence of many cells with the morphological appearance of apoptosis, such as a condensed nucleus surrounded by a halo. Consequently it can be concluded that 6-bromoisatin is the main factor in the *D. orbita* anticancer extracts contributing to enhancing the apoptotic response to the genotoxic insult of AOM. Although the effective doses of 6-bromoistain used in this study were high relative to common chemotherapeutic drugs, cancer prevention strategies are more likely to utilize dietary supplements or nutraceuticals containing higher doses of bioactive secondary metabolites with demonstrated low toxicity. 6-Bromoisatin is the dominant compound in oxidized extracts from the hypobranchial glands of *D. orbita*, an edible marine mollusc [40], This compound is stable at low pH in simulated digestive fluid [16] and appears to be bioavailable in the distal colon. Therefore, this paper supports the further development of a nutraceutical from Muricidae molluscs with potential application for the prevention of early stage colon cancer, by specifically targeting the 6-bromoisatin fraction.

## 3. Experimental Section

### 3.1. Synthetic 6-Bromoisatin and Chemical Analysis

Synthetic 6-bromoisatin (6-Bromoindole-2,3-dione) was purchased from TCI AMERICA (Portland, OR, USA) (purity of >97.0% GC). To confirm the identity and purity of the compound, ^1^H NMR spectroscopy (Bruker Avance III 400 MHz spectrometer, Preston, VIC, Australia) was performed in deuterated acetonitrile (Sigma Aldrich, Castle Hill, NSW, Australia). Chemical shifts (δ) are reported as parts per million (ppm) and referenced to residual solvent peaks. Spin multiplicities are indicated by: s, singlet; bs, broad singlet; d, doublet; t, triplet; q, quartet; m, multiplet; and dd, doublet of doublets.

### 3.2. *In Vitro* Experiments Using HT29 Colorectal Cancer Cells

All media and chemicals were purchased from Sigma Aldrich (Castle Hill, NSW, Australia) unless otherwise stated. HT29 human colorectal cancer cell line (passage no. 36-42) were cultured (37 °C and 5% CO_2_) in Dulbecco’s Modified Eagle’s Medium (DMEM) supplemented with 4500 mg/L l-glutamine, 10% FBS, 100 U/mL Penicillin/Streptomycin and 1% Non-essential Amino Acid (100×), until the cells reached 70% confluence.

The cells were harvested from flasks by trypsinization (1× Trypsin-EDTA) and were seeded (20,000 cells/well) into clear 96-well plates (Costar^®^, Mt Martha, VIC, Australia) for measurement of cell viability and white (opaque) 96-well plates (Interpath, Heidelberg West, VIC, Australia) for determination of apoptosis and necrosis. HT29 cells were incubated for 48 h, then the media was removed and the cells were washed with PBS. To treat the cells, synthetic 6-bromoisatin was dissolved in 100% dimethylsulphoxide (DMSO) then diluted in media and added to the cells at a range of concentrations from 0.1 to 0.01 mg/mL, in triplicate (final DMSO concentration of 1%). 1% DMSO controls were also included on each plate. Staurosporine (5 µM) for apoptosis and lysis solution (5 μL/well, Promega, Alexandria, NSW, Australia) for necrosis were added to the white plates in triplicate, as positive controls. All cells were treated for 12 h.

Morphological changes in the cells were observed on an Olympus CK2 inverted optical microscope (×400 magnification) 12 h after treatment. To measure cell viability, the MTT assay was applied, which measures the reduction of MTT tetrazolium salt to formazan, as previously described [15]. To measure necrosis and apoptosis, CytoTox-ONE Homogeneous Membrane Integrity Assay reagent (Promega) and Caspase-Glo 3/7^®^ assay (Promega) were applied respectively, according to our previous study [15]. These assays were all repeated in triplicate on three separate occasions (*n* = 3).

### 3.3. *In Vivo* Model for Early Stage Colon Cancer Prevention

This experiment was conducted under Flinders animal welfare approval number 751-10. A total of 38 wild-type (C57BL/6J) male mice aged 10 weeks were obtained from the Animal Resource Centre, Perth, Western Australia. Mice were divided randomly into 4 groups (ten mice in each treatment group and 8 mice in the sunflower oil only control group) and housed in 8 cages (four to five mice per cage). The mice were given water and food (rodent chow) *ad libitum* and maintained at the temperature of 22 ± 2 °C and humidity of 80% ± 10% with a 12 h light/dark cycle. Mice were weighed every five days and on the day of kill, and monitored daily for signs of illness, such as weight and hair loss, diarrhea, constipation, rectal bleeding, labored breathing, lethargy, eye and nose discharge.

To detect the early stage prevention of colon cancer, an established AARGC rodent model with injection of the carcinogen AOM was used [16,25,26,48]. Synthetic 6-bromoisatin at 3 different dosages (0.025, 0.05 and 0.1 mg/g body weight) was administered to mice by daily oral gavage in 100 µL sunflower oil, containing 0.02% Vitamin E, for two weeks. The control group was gavaged with sunflower oil (containing 0.02% Vitamin E) only. After two weeks, all mice were injected with a single intraperitoneal (i.p.) injection of AOM at a dosage of 10 mg/kg bodyweight and euthanized 6 h later by cervical dislocation under anesthesia. Our previous studies have shown that the peak time for the acute apoptotic response to carcinogen occurs between 6 h and 8 h post AOM injection (25), hence 6 h post AOM was chosen in the current study. The distal colon of each mouse was excised and fixed in 10% buffered formalin for 24 h and then embedded in paraffin for histological and immunohistological examination.

Distal colon segments were embedded in paraffin and sectioned at 4 µm (3–4 sections per mouse), then stained with hematoxylin, to evaluate apoptosis in epithelial cells of distal colon sections [48]. The slides were examined under a light microscope (Olympus BH-2, Mt Waverly, VIC, Australia, 400× magnification) to identify the apoptotic cells, by characteristic morphological changes such as cell shrinkage, condensed chromatin and sharply delineated cell borders surrounded by an unstained halo [26,49]. Twenty randomly chosen crypts were used to calculate the percent of apoptotic cells per crypt. The mean crypt column height was also determined.

Ki-67 is a cell cycle associated antigen and regarded as a useful proliferation marker [50]. Proliferative activity of distal colonic epithelial cells was measured using an antibody specific for the nuclear proliferating antigen ki-67 (rat-anti-mouse clone TEC-3, Dako, Campbellfield, VIC, Australia) in combination with an immunohistochemistry detection method in paraffin embedded sections, as previously described [15]. Sections of 4 µm were examined under a light microscope (Zeiss, Axio Imager A1, North Ryde, NSW, Australia) at 400× magnification to calculate the proliferation index as a percent of proliferated cells per crypt.

### 3.4. Liver Enzymes, Blood Biochemistry and Hematology

Blood samples (0.5–1 mL) were obtained from the mice under anesthesia by cardiac puncture at time of kill and transferred to Gribbles Veterinary Pathology laboratory, Adelaide within heparinized vacutainer tubes then centrifuged to separate into the cell layer for hematology analysis (Abbott Cell Dyn 3700 analyzer, North Ryde, NSW, Australia) and plasma for biochemistry analysis (Siemens Advia 1800 chemistry analyzer, Erlangen, Germany). The plasma levels of the liver enzymes AST, ALT and ALP were assessed as indicators of hepatotoxicity [39].

### 3.5. Statistical Analysis

Statistical analyses were performed using SPSS and values of *P* ≤ 0.05 were considered to be statistically significant. One way ANOVAs with post hoc Tukey HSD multiple comparisons were performed to determine which concentrations of 6-bromoisatin were significantly different to each other and the control.

## 4. Conclusions

In conclusion, this study supports the efficacy of synthetic 6-bromoisatin, at the concentration of 0.05 mg/g, for enhancing the apoptotic response to a genotoxic carcinogen and reducing cell proliferation in the distal colon of mice, without significant toxic effects detected in the liver or blood. The highest dose of 0.1 mg/g 6-bromoisatin showed a saturated dose–response for the induction of apoptosis, but had a stronger effect of inhibiting cell proliferation in the crypt of the distal colon and appears to also reduce the number of circulating white blood cells relative to the lower dose. Synthetic 6-bromoisatin appears to induce apoptosis in HT29 cells by a caspase-independent pathway. Although there was evidence of hypokalemia in the mice, due to the possible diuretic effect of 6-bromoisatin, no further toxicity in the liver or blood cells were observed. Longer term studies in mice are required to assess the effect of 6-bromoisatin on colonic aberrant crypt foci formation and/or tumor formation and also any possible side-effects associated with longer term use of this compound.
